# Incidence of A-V Fistulas after Renal Biopsy of Native and Transplanted Kidney - Two Centers Experience

**DOI:** 10.3889/oamjms.2015.049

**Published:** 2015-04-28

**Authors:** Mila Lubomirova, Rumiana Krasteva, Boris Bogov, Emil Paskalev

**Affiliations:** 1 *Department of Nephrology, University Hospital “Aleksandrovska”, Sofia, Bulgaria*; 2 *Department of Nephrology and Transplantation, University Hospital “Aleksandrovska”, Sofia, Bulgaria*

**Keywords:** renal biopsy, A-V fistula, Doppler ultrasound

## Abstract

**AIM:**

The aim of the study is to make a retrospective analysis of the incidence of AV fistulas after renal biopsy (RB) of native and transplanted kidney.

**MATERIALS AND METHODS:**

Five hundred and sixteen (516) RB were analyzed. One hundred twenty nine (129) were native kidneys RB performed in Clinic of Nephrology (CN), 190 were performed in Clinic of Nephrology and transplantation (CNT) and 197 were transplanted kidney biopsies from the same clinic. Biopsy technique type Gun with needle 14G, 16 and 18 G was used in CN, CNT used the same technique with needles 16G. Doppler ultrasound was made for A-V fistulas diagnosis.

**RESULTS:**

The A-V fistulas incidence was 0.8%. The frequency of A-V fistulas registered in CN was significantly higher than that registered in CNT (2.3% vs. 0.5%, p < 0.01). Biopsies performed by 14 G needles provide a higher percentage of A-V fistulas compared to those done by 16 G. (3.3% vs. 2.4%, p < 0.5). The frequency of the A-V fistulas in native and transplanted kidneys in CNT was similar (0.5% vs. 0.5%, p > 0.05).

**CONCLUSION:**

The A-V fistulas incidence is very low. The needle thickness is an important factor relevant to the risk of occurrence of A-V fistulas.

## Introduction

Renal biopsy (RB) is a routine procedure in Nephrology that allows a histological verification of the type of chronic kidney disease, and a definition of chronic kidney disease (CKD) activity as well as an evaluation of the disease activity, therapeutic behavior and a long term prognosis for the renal function [1-3]. This invasive manipulation hides certain risk of post biopsy complications [1-8]. Arterio-venous (A-V) fistula is a rare complication after RB. According to the literature its frequency after a native kidney biopsy varies from 0.3-5-6%, while in transplanted kidneys it can be up to 10-16% [4-15]. A-V fistulas after RB is a result of mechanical trauma cause by the biopsy needle to the arterial vessel wall and the collateral vena resulting in a communication between them. A-V fistulas can be visualized by Doppler examination. 70 % of A-V fistulas have no symptoms and are resorbed spontaneously within weeks. Less than 30% of A-V fistulas have clinical manifested with: high blood pressure, renal function deterioration, different degrees of macroscopic haematuria up to such that needs hemotransfusion, embolization or surgery [2, 14].

Volume overload of the heart as well as heart deficiency symptoms can appear with serious A-V fistula [8-20]. With Color Doppler fistula has a characteristic image: a mosaic patter, a mixture of blue and red colours. Pulse Doppler characteristics of the arterial vessel of the fistula are: low resistive index and high systolic and diastolic speed, while the venous vessel is arterialized with increased speed [1-3, 13-15, 18-20]. Angiography is the golden standard for making diagnosis [15-20].

The aim of the study is to summaries the experience to two Bulgarian leading nephrology centers about the incidence of A-V fistulas after RB of native and transplanted kidneys.

## Material and Methods

Five hundred and sixteen (516) PB of native and transplanted kidneys (380 male and 136 females biopsies) done within a period of 5.5 years were retrospectively analyzed. The average patient’s age is 45.3 ± 10 years. One hundred twenty nine (129) RB of native kidneys are done in Clinic of nephrology (CN) with in a period of 5 years, average patient’s age is 5 ± 1 years. One hundred ninety (190) RB of native kidneys are done in Clinic of Nephrology and Transplantation (CNT) within a period of 3 years, average patient’s age is 47.05 ± 22.03 years and 197 RB of transplanted kidneys are performed in the same clinic within a period of 8 years, average patient’s age is 38.05 ± 9.0 years. Glomerular filtration rate (GFR) measured by Cockroft - Gauld formula was used for renal function assessment. GFR < 90 ml/min was detected in 40 % of biopsied patients: 120 patients with kidney disease of native kidneys and 76 renal transplants, mean GRF 65.05 ± 32 ml/min.

All 516 renal biopsies (RB) were done according to individual criteria in absence of absolute contraindications after local anesthesia with Lydocain 2%. Native renal biopsy indications are: constant or recurrent hematuria, nephrotic or nephritic syndrome, unexplained renal function deterioration, acute kidney injury, hypertension of unknown cause, diabetes with suspicion for other disease overlay, systemic disease of connective tissue (lupus). Transplanted renal biopsy indications are: protocol biopsies, the appearance of all kinds of proteinuria, chronic or acute renal function deterioration, the presence of alloantibodies.

Blind renal biopsy technique is used and automated re-usable spring fired biopsy gun, with needle size 14, 16, 18 G - disposable Magnum, Bard system was performed in CN [12]. The biopsies were done by two qualified nephrologist who made more than 30 biopsies annually. Real time ultrasound control renal biopsy done by the automated re-usable spring fired biopsy gun, with needle size 16 G- disposable Magnum, Bard system was the method used in CNT in both native and kidney transplant [9, 12]. The biopsies were performed by one qualified nephrologist who performed more than 30 biopsies annually. The mean passes by which kidney tissue was obtained for histology examination in both biopsy techniques are two.

All patients are hospitalized and must keep standard regime after biopsy. Arterial pressure was monitories every 4 hours for a period of 48 hours. All patients were followed up for macroscopic hematuria for a period of 72 hours.

In order A-V fistulas to be diagnosed a Doppler examination of segmental and interlobar renal vessels were done in all RB up to 24 hours after the procedure [12]. Doppler ultrasound was done by Esaote Lab 60 machine. Doppler examination was performed by the nephrologist who made the biopsy.

Statistical software programs (SPSS 15.0.1) were used. All data were expressed as mean± SD. Statistical comparisons between two groups were made with a two–sample *t*- test. P < 0.05 was considered as statistically significant value.

## Results

About 99.5% of all biopsies contained more than 7 glomerulas are successful. Three out of 516 biopsies (0.5%) contain less than 7 glomerulas are considered unsuccessful. In 4 out of 516 biopsies A-V fistula was detected by Doppler ultrasound examination. We diagnosed 3 A-V fistulas after native biopsy and one after transplant renal biopsy. The A-V fistula frequency is 0. 8%. All 4 A-V fistulas are small, the mean size is 0.8 ± 0.89 mm and all they have no symptoms. The appearance of A-V fistula is not associated with an increased blood pressure, because the blood pressure before, during and 48 hours after the biopsy in all cases is not over 140/90 mmHg. Spontaneous disappearance of all 4 A-V fistulas is diagnosed by Doppler within one month after biopsy.

The incidence of A-V fistulas after native kidney biopsy in CN is 2.3% and is significantly higher as compared to those in CNT (Table 1).

**Table 1:** Native kidney biopsy A-V fistulas incidence.

Clinical Center	A-V fistula
Native RB- CNT	1/190, 0.5%

Native RN- CN	3/129, 2,3%

P	<0.01

The native renal biopsy technique performed in CN is different from that used in CNT. In CN blind RB is performed, using needles of three different sizes- 14, 16, 18G. Two of the three A-V fistulas in native kidneys in Clinic of Nephrology are derived from the thick needles- 14 G. Biopsies done by 14G needles give significantly higher percentage of A-V fistulas compared to those done by 16G needles (3.3% vs. 2.4%, p < 0.01). Thin needles- 18G do not give fistulas (Table 2).

**Table 2:** The frequency of A-V fistulas after native RB in CN according to the needle size.

Needle size	A-V fistula
18 G- 10 RB	0

16 G – 89 RB	2 /2.4%

14 G- 30 RB	1 /3.3%

Total number of RB 129	3 /2.3 %

The frequency of A-V fistulas after native and transplanted RB in CNT is equal- one case in transplant kidney (1 out of 190 biopsies) and one after native kidney biopsy (1 out of 197 biopsies) - 0.5% vs. 0.5%. In these cases only real time ultrasound control renal biopsy done by the automated re-usable spring fired biopsy gun, with needle size 16 G was used.

## Discussion

This is the first retrospective study to summaries the frequency of the A-V fistulas after on native and transplanted kidneys RB done in two of the biggest Nephrology centers in Bulgaria. The asymptomatic A-V fistulas frequency, reported in our study, is very low- 0.8% and is commensurate with the results published during the last 10 years [1-20]. According to the literature e the incidence of post biopsy A-V fistulas of native kidneys vary from 0. 3- 6-10 % even to 16 % depending mostly on the technical parameters of the manipulation. To determine the incidence of A-V fistulas is important who carried out the post- biopsy Doppler control [1,16,17]. Our recent studies have proved the low frequency of the post biopsy A-V fistulas [2-3]. The clinical manifestation of A-V fistulas is highly varied and depends on the size, localization, blood flow speed and whether this pathological communication between artery and vein increases with the time or is resorbed and spontaneously disappears. Big A-V fistulas are likely to have no symptoms [14-20]. The time of the post biopsy trauma fistula appearance and its clinical manifestation vary within broad frames- the symptoms may show up months after RB which leads to the conclusion that once having been proved the post-biopsy A-V fistula must be systematically examined by renal vessels Doppler ultrasound months after the RB [15-20]. We detect all 4 A-V fistulas after renal biopsies in both native and transplanted kidney within 24 hours after biopsy and all they disappeared within a month after biopsy without symptoms. In 2012 Copari K and co-authors made a meta- analysis of more than 2472 abstracts and referred 299 articles and more than 8000 biopsies in order to establish the frequency of the various post- biopsy complications. The team analyzed only one randomized controlled study on that subject, all the rest being retrospective and done by one center. An A-V fistula frequency under 3% was also announced – a result similar to our data [7]. This meta-analysis and our experience allows us to conclude that RB of native and transplanted kidneys is an invasive manipulation which carries a minimum risk of post biopsy A-V fistulas appearance [1-20]. The various results of the various studies are explained by various technical characteristics of the RB used, the various needle sizes, the type of biopsy technique as well as the various methods and terms of control. Using one and same biopsy technique in CNT the frequency of post- biopsy fistulas of native and transplanted kidneys show no difference. Similar data are published in other studies [1-20]. The higher frequency of the A-V fistulas on native kidney performed in CN is explained by the biopsy technique used- blind biopsy as well as the utilization of needles of different size. The subject of this article is only to define the post- biopsy A-V fistulas frequency not taking in mind the factors related to the risk of complications- for example, age, renal function, urgency of the procedure, BMI etc. If these factors are analyzed, out of the technical parameters of the biopsy technique no doubt there will be a more complex explanation for causes of the appearance of these complications. This first article leads to the conclusion that common effort is necessary for establishing a RB register and the register of post-biopsy complications. Our results make as conclude that the technical parameters of the biopsy technique are explicitly related to the risk of A-V fistula appearance. Biopsy done with 16 G needles has an advantage over the quality of the material taken- the material is considerably bigger which is of substantial clinical importance. The optimum period for ultrasound follow up after RB for A-V fistulas diagnosis is up to 24-th hour after the manipulation. Summarizing the literature data and our results a conclusion can be made that the renal biopsy technique is of great importance for diminishing the risk of A- V fistulas incidence.

We can conclude that the gold standard for biopsy technique is: renal biopsy under ultrasound control done by the automated re-usable spring fired biopsy gun, with needle size 16 G. Despite possible post- biopsy complications the kidney biopsy is a routine invasive manipulation in Nephrology which puts patients in a minimum risk and is necessary for the diagnostic and therapeutic process.
